# Dominance in Habitat Preference and Diurnal Explorative Behavior of the Weakly Electric Fish *Apteronotus leptorhynchus*

**DOI:** 10.3389/fnint.2019.00021

**Published:** 2019-07-05

**Authors:** Till Raab, Laura Linhart, Anna Wurm, Jan Benda

**Affiliations:** Institute for Neurobiology, Eberhard Karls Universität, Tübingen, Germany

**Keywords:** animal behavior, weakly electric fish, dominance, diurnal activity, habitat selection

## Abstract

Electrocommunication and -localization behaviors of weakly electric fish have been studied extensively in the lab, mostly by means of short-term observations on constrained fish. Far less is known about their behaviors in more natural-like settings, where fish are less constrained in space and time. We tracked individual fish in a population of fourteen brown ghost knifefish (*Apteronotus leptorhynchus*) housed in a large 2 m^3^ indoor tank based on their electric organ discharges (EOD). The tank contained four different natural-like microhabitats (gravel, plants, isolated stones, stacked stones). In particular during the day individual fish showed preferences for specific habitats which provided appropriate shelter. Male fish with higher EOD frequencies spent more time in their preferred habitat during the day, moved more often between habitats during the night, and less often during the day in comparison to low-frequency males. Our data thus revealed a link between dominance indicated by higher EOD frequency, territoriality, and a more explorative personality in male *A. leptorhynchus*. In females, movement activity during both day and night correlated positively with EOD frequency. In the night, fish of either sex moved to another habitat after about 6 s on average. During the day, the average transition time was also very short at about 20 s. However, these activity phases were interrupted by phases of inactivity that lasted on average about 20 min during the day, but only 3 min in the night. The individual preference for daytime retreat sites did not reflect the frequent explorative movements at night.

## 1. Introduction

Weakly electric fish are nocturnally active. In the night, many pulse-type fish increase the rate of their electric organ discharges (EOD) (Lissmann and Schwassmann, 1965; Stoddard et al., 2007), wave-type fish emit various kinds of electrocommunication signals more frequently (Zupanc et al., 2001; Henninger et al., 2018), and gymnotids have been shown to move from deep waters up to the shore (Steinbach, 1970). During the day, weakly electric fish hide under submerged logs (*Gymnotus*, Westby, 1988), between roots (*Eigenmannia*, Hopkins, 1974), in leaf litter (*Brachyhypopomus*, Hagedorn, 1988), or bury themselves in sand (*Gymnorhamphichthys*, Lissmann and Schwassmann, 1965).

EOD frequencies of the gymnotiform brown ghost knifefish *A. leptorhynchus* are sexually dimorphic with males having higher EOD frequencies than females (Meyer et al., 1987). In playback experiments with restrained fish, males more frequently produced aggressive communication signals (chirps) than females (Zupanc and Maler, 1993; Bastian et al., 2001) and in experiments of free swimming fish, male *A. leptorhynchus* showed a higher overall chirp rate compared to females (Dunlap and Oliveri, 2002; Hupé and Lewis, 2008). However, during courtship in the field females produced almost as many chirps as males (Henninger et al., 2018), and both sexes jammed rivals by approaching their EOD frequencies (Tallarovic and Zakon, 2002). In competition experiments, male *A. leptorhynchus* were more likely to inhabit tubes alone, whereas females cohabited tubes more often (Dunlap and Oliveri, 2002).

Several studies suggest higher EOD frequencies in males as an indicator of dominance. Additionally, body size correlated with EOD frequency in males (Triefenbach and Zakon, 2008; Fugère et al., 2011) but not in females (Dunlap and Oliveri, 2002). Dominant males with higher EOD frequencies were more aggressive (Fugère et al., 2011) and participated more in mating (Hagedorn and Heiligenberg, 1985; Henninger et al., 2018). In competition experiments, males with higher EOD frequencies occupied the most preferred tubes, whereas females did not distribute according to EOD frequency (Dunlap and Oliveri, 2002). In summary, these laboratory studies suggest that male brown ghost knifefish are territorial at their preferred retreat site during the day, and that males with higher EOD frequencies are more dominant.

Observations on aggression and dominance have previously been limited to studies in the lab in small tanks, and mostly to short observation times (e.g., Hopkins, 1974; Hagedorn and Heiligenberg, 1985; Nelson and MacIver, 1999; Tallarovic and Zakon, 2005; Hupé and Lewis, 2008; Triefenbach and Zakon, 2008). Recent technological advances allow for long-term observations of electric activity of these fish in the lab and in the field (Henninger et al., 2018; Madhav et al., 2018). Here, we take advantage of these methods and describe diurnal activity patterns of a community of *A. leptorhynchus* competing for different microhabitats in a large indoor tank over 10 days.

## 2. Methods

**Figure 1:**
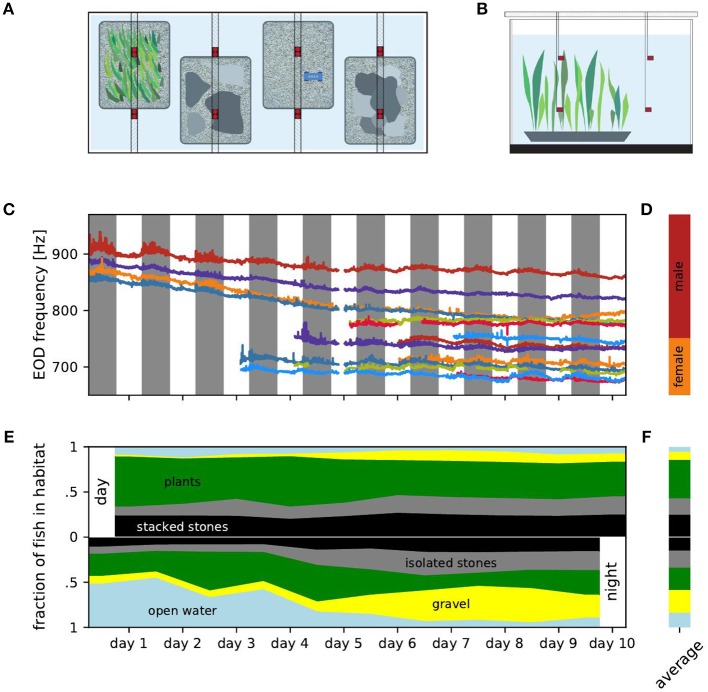
Experimental setup, EOD frequencies and distribution of fish over habitats. **(A)** Top view of the experimental setup with four different micro habitats (plants, isolated stones, gravel, stacked stones). Electrodes (red) were fixed in location by PVC poles positioned above the tank. Fish were fed on a daily basis on the gravel habitat using a custom PCV feeder (blue). **(B)** Side view of the experimental setup showing the electrodes positioned in two levels over the habitats. **(C)** EOD frequency traces tracked over the entire duration of the experiment. Individual fish are marked by the same color in all figures. **(D)** Ranges of male (red) and female (orange) EOD frequencies. **(E)** Fraction of fish detected within each of the five habitats for consecutive days (top) and nights (bottom). **(F)** Relative occupation of the habitats averaged over all days (top) and nights (bottom).

Six male and eight female *A. leptorhynchus*, obtained from a tropical fish supplier, were housed in a 2.5 × 1 × 0.8 m^3^ indoor tank with a water conductivity of 320 μS/cm at a 12 h/12 h light cycle. Initially, four fish inhabited the tank. Starting at day 4 we introduced two additional fish per day. Fish were selected for approximately equal size to minimize effects based on physical differences as far as possible. All fish were mature and not in breeding condition. EOD frequency is sexually dimorphic in *A. leptorhynchus* (Meyer et al., 1987). We identified fish with EOD frequencies lower than 750 Hz as females, and fish with higher EOD frequencies as males (Henninger et al., 2018). Four natural-like habitats in 60 × 45 × 10 cm^3^ PVC-containers were arranged next to each other in the tank: stacked stones, quartz gravel (few millimeters diameter), isolated stones, and aquatic plants (*Vallisneria spec*.) (Figure 1A). Fish were fed frozen *Chironomus plumosus* on the gravel habitat every day at about 8 h after lights were switched on. Animal housing complied with national and European law and was approved by the Regierungspräsidium Tübingen (permit no: 35/9185.46/UniTÜ). Approval by an ethics committee was not required because our study was purely observational.

We continuously recorded EODs for 10 days and nights using 16 monopolar electrodes at low-noise headstages, and digitized at 20 kHz per channel with 16 bit resolution (see Henninger et al., 2018 for details). For each of the four habitats, two electrodes were placed at the bottom of the habitat 35 cm apart and two electrodes 35 cm above the respective electrodes in the habitats in the open water (Figures 1A,B). Water temperature was measured once a day. During the course of the experiment, water temperature steadily dropped from 26.3°C to 24.8°C. Fish were identified by their specific peaks in the spectrogram of the recordings (nfft = 2^16^, overlap = 90%) and tracked using a custom tracking algorithm comparing fundamental EOD frequency and the corresponding power pattern in the spectrograms of the different electrodes (see Henninger et al., 2018 and Madhav et al., 2018 for details).

**Figure 2:**
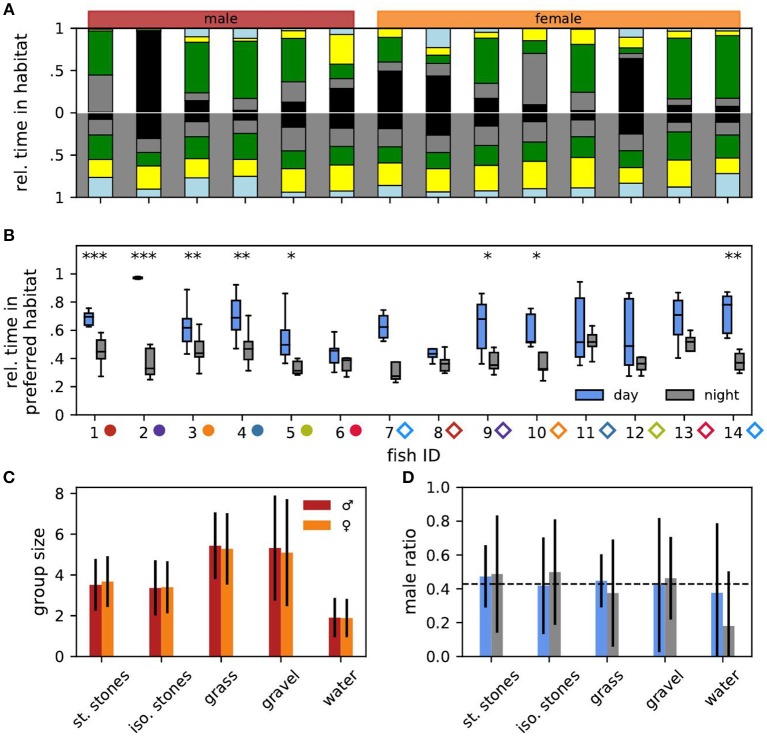
Habitat preference. **(A)** Relative time each individual fish spent in the different habitats (same color code as in Figure 1E) averaged over all days (top) and nights (bottom). Males (fish IDs 1–6) are indicated in red, females (fish IDs 7–14) in orange. Male and female fish IDs are sorted according to descending EOD frequency in all figures. **(B)** For each fish and day (blue) or night (gray) the fraction of time the fish spent in its currently preferred habitat. Asterisks indicate significant differences: ^***^p < 0.001, ^**^p < 0.01, and ^*^p < 0.05. **(C)** For each habitat the mean group size with standard deviation in which males (red) and females (orange) were found after the maximum of 14 fish had been reached. **(D)** For each habitat the average male ratio with standard deviation during the day (blue) and night (gray) after the maximum of 14 fish had been reached.

Every 0.328 ms (temporal resolution of the spectrogram), fish were assigned to habitats by means of the electrode with the largest power at the fish's EOD frequency. Based on this spatial information we analyzed how the fish occupied the habitats. For each day and night, we computed the fraction of fish in each habitat by dividing the detections within one habitat by the total number of detections on that day or night (Figure 1E). Likewise, individual habitat preferences were computed separately based on the detections of each fish (Figure 2A). To assess the number and composition of fish in each habitat we counted the number of males and females detected in each habitat for every time step (Figure 2C). The male ratio is the number of males in a habitat divided by the total number of detected fish in that habitat (Figure 2D).

**Figure 3:**
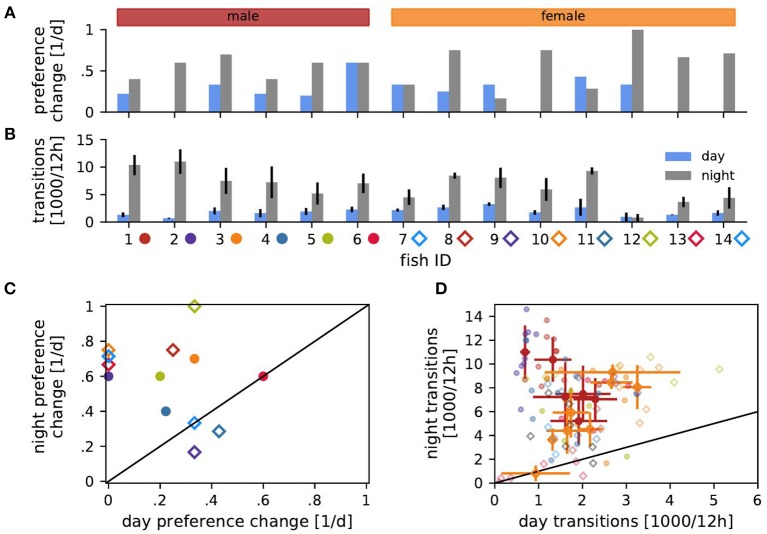
Transitions of habitat preference and between habitats. **(A)** Probability of changing the preferred habitat from one day (blue) or night (gray) to the next for each fish. **(B)** Transition rates, i.e., number of detected transitions between habitats per 12 h, averaged over days (blue) or nights (gray) with standard deviation. **(C)** Probabilities of changing preference of night habitats vs. preference changes of day habitats from **(A)**. **(D)** Transition rates during the day vs. transition rates at night from **(B)**. Transition counts averaged over days and nights with standard deviation are shown for each male (red) and female (orange). Symbols in **(C,D)** indicate fish ID as in **(B)**.

The preferred habitat of a fish was defined as the habitat where the fish spent most of the time, i.e., had the most detections, for each day and night. Relative time spent in the preferred habitat was computed as the ratio between detections in the preferred habitat and the number of detections per day or night (12 h × 3,600 s/h × 3.05 detections per second ≈ 131,827 detections per 12 h) for every day and night (Figure 2B). The stability of individual habitat preferences were evaluated using preference change rates, i.e., the probability of a fish to change its preferred habitat from one day or night to another one, computed as the number of days (or nights) on which the fish preferred a different habitat as on the previous day (or night) divided by the number of days the fish was in the tank minus one (Figures 3A,C).

**Figure 4:**
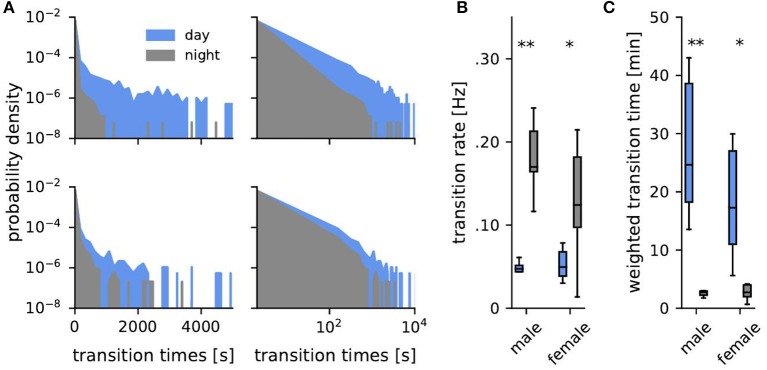
Transition times. **(A)** Probability density of transition times (time span spent consecutively within one habitat) during the day (blue) and the night (gray) for one example male (top, fish ID 4) and female (bottom, fish ID 10). **(B)** Corresponding transition rates obtained from fitting an exponential to the distributions of transition times. **(C)** Averaged weighted transition times (Equation 2). Asterisks indicate significant differences: ^**^p < 0.01 and ^*^p < 0.05.

Transitions of fish between habitats were characterized by the number of transitions of detections from electrodes of one habitat to electrodes from another habitat (Figures 3B,D). The distributions of transition times Δ*t*, i.e., the time spans a fish spent in one habitat between two habitat changes, were exponentially distributed (Figure 4A):

(1) $$\begin{array}{l} p\left(\Delta t\right)=\lambda e^{-\lambda \Delta t}. \end{array}$$

The number of transitions per time (Figure 3B) is the transition rate. In Figure 4B the transition rate λ = 1/τ was estimated from the average transition time $\tau =\frac{1}{n}\overset{n}{\underset{i=1}{\sum }}\Delta t_{i}$ for each fish separately for days and nights.

The tails in the distributions of transition times dominate the activity patterns of the fish because a single long transition time implies a non-moving fish for exactly this time. During the same time, however, many more short transitions can occur. Short transition times are thus overrepresented when taking the average. To account for this we also computed a weighted average $\bar{\Delta t_{i}}$, where we weighted each transition time Δ*t*_*i*_ by its duration Δ*t*_*i*_ (Figure 4C):

(2) $$\begin{array}{l} \bar{\Delta t_{i}}=\frac{\sum_{i=1}^{n}\Delta t_{i}^{2}}{\sum_{i=1}^{n}\Delta t_{i}} \end{array}$$

Finally, we investigated if individual habitat changes were independent of each other by calculating the time differences between a fish entering a habitat and the other fish leaving the respective habitat. We compared these distributions to boot strapped distributions where entering times to a random habitat were set randomly throughout the whole recording period.

Because fish were in similar physical condition and their sexes were determined using only a hard EOD frequency cutoff at 750 Hz we performed a sensitivity analysis for all corresponding results, i.e., additionally to the original sex assignments, all statistics were calculated with up to ±2 males or females, where the individuals closest to the cutoff were assigned to the opposite sex.

For quantifying differences between groups we used Cohen's *d* for unequal group sizes:

(3) $$\begin{array}{l} d=\left|\frac{\mu_{1}-\mu_{2}}{\frac{n-1}{n+m-2}\sigma_{1}^{2}+\frac{m-1}{n+m-2}\sigma_{2}^{2}}\right| \end{array}$$

where μ_1_ and μ_2_ are the means, σ_1_ and σ_2_ the standard deviations, and *n* and *m* the group sizes, respectively.

## 3. Results

We observed the movements of six male and eight female *A. leptorhynchus* between four microhabitats and the open water in a two cubic meter tank over 10 days. We tracked individual fish based on EOD frequency and power on 16 recording electrodes (Figure 1C). EOD frequency is known to be sexually dimorphic in *A. leptorhynchus* (Meyer et al., 1987). Fish with an EOD frequency above 750 Hz are defined as males, fish below 750 Hz as females (Figure 1D, Henninger et al., 2018). The overall decline of EOD frequencies followed the water temperature, which decreased by 1.5°C over the course of the experiment. In fact, the *Q*_10_ values computed for each fish from daily temperature measurements and the corresponding EOD frequencies (median *Q*_10_ = 1.54) were close to typical *Q*_10_ values reported for these fish in the literature (Dunlap et al., 2000; Stöckl et al., 2014). Additionally, circadian modulations of each fish's EOD frequency followed similar patterns and can also be best explained by periodic diurnal water temperature changes (Dunlap et al., 2000). On top of these exogenous influences, the fish actively changed their EOD frequency, approaching and evading EOD frequencies of other fish. For example, the EOD frequencies of the males indicated in orange and blue approached each other and got closer to the males indicated in red and green. Female fish also approached each other in their EOD frequency and even switched order (e.g., the females indicated in red and light blue at the bottom of Figure 1C). In the following we do not analyze these modulations of EOD frequency but rather focus on diurnal movement patterns.

### 3.1. Habitat Occupation

The tank offered the fish four different 0.25 m^2^ habitats that contained either stacked stones, quartz gravel, isolated stones, or aquatic plants. We counted the open water above the habitats as a fifth habitat. For each time point we assigned each fish to one of these habitats according to the electrode with the largest power at its EOD frequency.

During the days, i.e., the presumably inactive phases of the fish, most fish were located within the aquatic plants followed by the stacked stones and the isolated stones. Fish were rarely found in the gravel habitat or in the open water (Figures 1E,F, top). At night, no habitat seemed to be preferred on average (Figures 1E,F, bottom).

During the days, the addition of fish did not influence the distribution of fish in the habitats by a lot (Figure 1E, top). The standard deviation of the fraction of fish occupying a habitat was below 6.5 % for all habitats. Nevertheless, the fraction of fish occupying isolated stones or gravel increased slightly throughout the experiment (Spearman's rank correlation: *r* = 0.76, *p* = 0.005 and *r* = 0.88, *p* < 0.001, respectively), whereas the occupation of the aquatic plants and the open water slightly decreased within the 10 days (Spearman's rank correlation: *r* = −0.76, *p* < 0.05 and *r* = −0.65, *p* < 0.05, respectively). The occupation of the stacked stones habitat was unaffected by the increasing fish count and did not change over the days of the experiment (Spearman's rank correlation: *r* = 0.37, *p* = 0.30). Consequently, the increasing total fish count lead to an almost uniform increase in the number of fish occupying each habitat. None of the habitats was claimed exclusively by a dominant fish as a retreat site during the days.

In contrast, at nights the increased fish count seemed to influence the distribution of the fish over the habitats more strongly (Figure 1E, bottom). The occupation of both the isolated and stacked stones habitats increased slightly during the course of the experiment (Spearman's rank correlation: *r* = 0.84, *p* < 0.01 and *r* = 0.79, *p* < 0.01, respectively), whereas the fraction of fish in the open water clearly decreased (Spearman's rank correlation: *r* = − 0.90, *p* < 0.001) and the occupation of the gravel habitat increased (Spearman's rank correlation: *r* = 0.95, *p* < 0.001). The latter could be attributed to the experimental design. Food was supplied daily at the gravel habitat and gymnotiform fish have been shown to learn the location of food (Jun et al., 2013).

### 3.2. Habitat Preferences

Let us now turn to the habitat preferences of individual fish (Figures 2A,B). Even during the day fish did not stay at the same habitat. Male no. 2 was the only exception, which throughout the experiment stayed in the stacked stones at daytime (Figure 2A, top). The preferred daytime habitat, i.e., the habitat the fish stayed the longest during the day, varied between individuals. Some fish preferred the stacked stones, whereas others preferred the isolated stones or the plants. Only male no. 6 had a slight preference for the gravel habitat. In the night, individual fish had less obvious preferences for specific habitats on average (Wilcoxon: *W* = 0, *p* = 0.001, Figure 2A, bottom).

The fish sometimes changed their preferred habitat from one day to another (Figure 3A). Preferred nighttime habitats were changed more often than daytime habitats (Wilcoxon: *W* = 3, *p* = 0.005) (Figure 3C). The probability of changing the preferred habitat from one day to the next did not significantly correlate with EOD frequency, neither for males nor for females (Spearman's rank correlation: *p* > 0.2).

In particular males stayed significantly longer in their preferred daytime habitat than in their preferred nighttime habitat (Figure 2B). Furthermore, males with higher EOD frequencies spent more time in their preferred daytime habitat than low-frequency males (Spearman's rank correlation: *r* = 0.49, *p* < 0.001). For males at night and females no such correlation was significant (Spearman's rank correlation: *p* > 0.1).

To summarize, with the exception of male no. 2, the notion of a “preferred habitat” turns out to be misleading. Of course there is always a habitat where a fish spends most time during a day or night simply by definition. However, other habitats are visited as well (Figures 2A,B) and even the preferred habitat is changed within a few days (Figure 3A).

### 3.3. Group Sizes and Composition

Many fish had similar habitat preferences. This should be reflected in the number of fish found in each habitat. For quantifying group sizes and compositions in the different habitats we analyzed the final 78 h where all fourteen fish were present in the tank. The mean group size differed between the habitats (Figure 2C). Significantly less fish were simultaneously detected in the open water (1.89 ± 0.95) than in the isolated stones (3.39 ± 1.32, Mann-Whitney U: *p* < 0.001, Cohen's *d* = 1.20), and stacked stones (3.61 ± 1.26, Mann-Whitney U: *p* < 0.001, Cohen's *d* = 1.43). Group sizes in the gravel (5.21 ± 2.61) and plant habitat were significantly larger than in both stone habitats and the open water (Mann-Whitney U: *p* < 0.001, Cohen's *d*: 0.78 < *d* < 2.16).

Interestingly, male ratios in all habitats were close to the expected 0.43 given by the overall number of six males and eight females (dashed line in Figure 2D, Cohen's *d* < 0.24). There was no difference in habitat preferences between the sexes. Only in the open water at night the male ratio was considerably lower than expected (Cohen's *d* = 0.77).

### 3.4. Transitions Between Habitats

Fish frequently moved between habitats (Figure 3B). The EOD frequency of males correlated negatively with the number of transitions between habitats during the day and positively during the night (Spearman's rank correlation: *r* = −0.47, *p* < 0.01 and *r* = 0.55, *p* < 0.001, respectively). That is, high-frequency males were more territorial during the day and more explorative at night than low-frequency males. In females, transition counts correlated positively with EOD frequency during both day and night (Spearman's rank correlation: *r* = 0.55, *p* < 0.001 and *r* = 0.45, *p* < 0.01). Therefore, females with higher EOD frequency were more active.

Both males and females switched habitats significantly more often during the night than during the day (Figure 3D). The more stationary males were during the day, the more explorative they were at night (Spearman's rank correlation: *r* = −0.49, *p* < 0.001). On the other hand, female transition counts during day and night were positively correlated (*r* = 0.53, *p* < 0.001). No such correlations existed for individual fish.

Transition times, i.e., the time intervals between habitat transitions, were approximately exponentially distributed (Equation 1, Figure 4A). Such exponential distributions are generated by Poisson point processes where the probability of an event (here a transition to another habitat) is the same for each time point and independent of previous events, like for example radioactive decay or state transitions of ion channels. There was no distinguished time scale that separated activity phases from resting phases. Transition rates (Figures 3B, 4B) were generally quite high and average to 0.1 Hz. They were significantly larger during the night than during the day for both, males (Mann-Whitney U: *U* = 0, *p* < 0.01, *d* = 4.05) and females (Mann-Whitney U: *U* = 8, *p* < 0.05, *d* = 1.58), and were independent of sex (Figure 4B).

Averaged weighted transition times, Equation (2), better capture differences on long time scales, reflecting non-moving fish. On average weighted transition times were 20 min during the day and 3 min at night (Figure 4C, Mann-Whitney U: males *U* = 0, *p* < 0.01, *d* = 1.41, females *U* = 8, *p* < 0.05, *d* = 0.34).

Transitions of individual fish were independent from other fish entering the habitat (not shown). The distribution of times between a fish entering a habitat and another fish leaving the same habitat showed statistically significant (Kolmogorov-Smirnov test, *p* < 0.001) but small differences to a distribution generated for times of a fish entering a randomly chosen habitat drawn from a uniform distribution (Cohen's *d*: 0.02 < *d* < 0.08).

### 3.5. Sensitivity Analysis

Since we based the sex of the fish on EOD frequency only, we repeated all analysis for different EOD frequency thresholds separating males from females. For up to two males reassigned to females and vice versa the sex dependent results in the contexts of Figures 3C,D, 4B,C did not change. All significant levels as well as effect sizes stayed in the same range.

## 4. Discussion

We observed movement patterns and habitat preferences in a population of fourteen brown ghost knifefish, *A. leptorhynchus*, in a large indoor tank over 10 days. During the day, these nocturnal fish distributed themselves quite uniformly in habitats providing appropriate retreat sites between stones or plants. Activity at night was characterized by strong explorative movements where fish frequently changed between habitats and the open water. In male fish, high EOD frequency correlated with more territorial behavior during days and a more explorative personality at night, whereas in female fish EOD frequency was positively correlated with movement activity during both day and night.

### 4.1. Nocturnal Activity

Despite the well supported common notion of weakly electric fish being nocturnally active (Lissmann and Schwassmann, 1965; Zupanc et al., 2001; Henninger et al., 2018), our data show that phases of activity, as indicated by short transition times between the habitats, occurred in similar ways both at night and during the day (Figure 4A). There was no qualitative difference between day and night. During the day, phases of inactivity were prolonged about ten-fold in comparison to the ones at night (Figures 4B,C). Otherwise, activity, as quantified by transitions between habitats, occurred randomly and independently of each other. This fits well with the description of stochastic onsets of activity phases in *Gymnotus* (Jun et al., 2014).

### 4.2. Retreat Site Selection

Selection of an appropriate retreat site has profound effects on the animal's physiological condition and fitness (Rosenzweig, 1981; Huey, 1991). All of the preferred retreat sites in our experiment offered appropriate places where fish could hide. This fits well to field observations where fish were also found hiding under submerged logs, between roots, or in leaf litter during the day (Hopkins, 1974; Hagedorn, 1988; Westby, 1988). Our data demonstrates that, at least in captivity, most fish do not depend on specific retreat sites, like for example stacked stones, but rather change between many available types of microhabitats.

In small tanks in the laboratory males often compete over tubes provided for refuge (Hopkins, 1974; Hagedorn, 1988; Fugère et al., 2011). In the presence of enough tubes, male *A. leptorhynchus* preferred to occupy tubes alone, but females were sometimes found together in single tubes (Dunlap and Oliveri, 2002). Fish had clear preferences when presented with a variety of tubes of different dimensions and opacity (Dunlap and Oliveri, 2002).

In our study fish showed individual preferences for different habitats (Figure 2A). The grass and gravel habitat accommodated the most individuals simultaneously, and the open water the least (Figure 2C). This indicates either differences in general habitat quality or differences in the actual number of available suitable retreat sites in each of the habitats. The fraction of males found in each habitat on average did not deviate from the expectation given the total number of males and females (Figure 2D). Thus, group composition on the scale of a whole habitat was not influenced by the hierarchical status of individual fish. However, our experimental design did not allow to resolve group compositions on a finer spatial scale of specific retreat sites within each habitat. Our data therefore do not contradict an influence of hierarchical status on retreat site selection as reported by Dunlap and Oliveri (2002).

### 4.3. Social Dominance

The EOD and its modulations convey information about species, sex, status and intent of individuals (e.g., Hagedorn and Heiligenberg, 1985; Stamper et al., 2010; Fugère et al., 2011). In *A. leptorhynchus* EOD frequency correlates with body size (Dunlap, 2002; Triefenbach and Zakon, 2003). Furthermore, dominant males in breeding contexts in the laboratory (Hagedorn and Heiligenberg, 1985) as well as in the field (Henninger et al., 2018), and in tube selection contexts (Dunlap and Oliveri, 2002; Fugère et al., 2011) had higher EOD frequencies. We here reported a more subtle variant of dominance. Male fish with higher EOD frequency moved less between habitats during the day and showed increased movement activity at night compared to males with lower EOD frequency. These increased nocturnal movement activities could reflect frequent fights for dominance (Tallarovic and Zakon, 2005), as the approaching EOD frequencies of the fish suggest (Figure 1C). Contrary to the expectation of fish fighting for dominance, the time points of fish leaving a habitat were independent from fish entering the respective habitat. A closer inspection of the EOD frequency traces for communication signals like rises and chirps (Zakon et al., 2002) could help classify different types of movement activities and interactions in the future (Triefenbach and Zakon, 2008). In females, EOD frequency did not appear to be correlated with dominance (Dunlap and Oliveri, 2002). However, we found that EOD frequencies of females were positively correlated with movement activity during both day and night. Rather than an indication of hierarchical status, EOD frequency seems to indicate individual activity personalities (Sih et al., 2004).

## 5. Conclusion

Many laboratory studies on the behavior of weakly electric fish focused on specific questions that were tested in temporally and spatially limited experimental settings (Hopkins, 1974; Hagedorn and Heiligenberg, 1985; Nelson and MacIver, 1999; Tallarovic and Zakon, 2002; Hupé and Lewis, 2008; Triefenbach and Zakon, 2008). Recent advances in recording techniques (Henninger et al., 2018; Madhav et al., 2018) allowed us to continuously monitor a population of weakly electric fish in a large tank with a more natural-like setting for many days. In particular, we did not force the fish into specific behaviors, but rather, extracted behavioral activity patterns from the data (Gomez-Marin et al., 2014). In this way, we revealed personality like differences in territoriality and explorative movements (Sih et al., 2004). In both males and females these were correlated with EOD frequency, suggesting EOD frequency as an indicator for more explorative personalities in both sexes, and territoriality in males.

## Data Availability

The datasets generated for this study are available on request to the corresponding author.

## Ethics Statement

Pure observational study on weakly electric fish.

## Author Contributions

TR: designed the experiment, analyzed data, and wrote the manuscript. LL and AW: measured and analyzed the data. JB: discussed the experiment and advised data analysis and wrote the manuscript.

### Conflict of Interest Statement

The authors declare that the research was conducted in the absence of any commercial or financial relationships that could be construed as a potential conflict of interest.
